# Presurgical Weight Is Associated with Pain, Functional Impairment, and Anxiety among Gastric Bypass Surgery Patients

**DOI:** 10.1155/2012/412174

**Published:** 2012-10-15

**Authors:** Sharlene Wedin, Karl Byrne, Katherine Morgan, Marie LePage, Rachel Goldman, Nina Crowley, Sarah Galloway, Jeffrey J. Borckardt

**Affiliations:** Medical University of South Carolina, 67 President Street, IOP 1-South, Suite 104, Charleston, SC 29425, USA

## Abstract

Chronic pain and obesity are significant public health concerns in the United States associated with significant levels of health-care expenses and lost productivity. Previous research suggests that obesity is a risk factor for chronic pain, mainly due to excessive weight placed on the joints. However, the obesity-pain relationship appears to be complex and reciprocal. Little work to date has focused on the relationship between weight and pain among patients undergoing gastric bypass surgery for weight loss. Patients scheduled to undergo bariatric surgery for weight loss at a large southeastern academic medical center (*N* = 115) completed the Brief Pain Inventory (BPI), the Center for Epidemiological Studies 10-item Depression scale (CESD-10), and the Beck Anxiety Inventory (BAI). Higher presurgical weight was associated with higher pain-on-average ratings, higher functional impairment due to pain across the domains of physical activity, mood, walking ability, relationships, and enjoyment of life. Higher presurgical weight was associated with higher BAI scores, but weight was not related to depression. Findings suggest that bariatric surgery candidates report a moderate amount of pain prior to surgery and that presurgical weight is associated with higher pain, increased functional impairment due to pain, and increased anxiety. Anxiety was found to mediate the relationship between increased weight and pain.

## 1. Introduction

Chronic pain and obesity are significant public health concerns in the United States, with costs related to obesity estimated to be at $118 billion annually, and chronic pain is associated with $70 billion a year in health-care expenses and lost productivity. While an obesity-pain relationship would seem quite apparent, only recently has the obesity-pain association been confirmed in a large-scale population based study [1]. 

Previous research suggests that obesity is a risk factor for chronic pain, mainly due to excessive weight placed on the joints. One such study examined the relationship between body mass index (BMI) and reports of significant knee, hip, and back pain in a large sample of older adults. They found that reported pain increased with higher BMIs for all types of pain measured [2]. However, the pain-obesity relationship extends beyond the lower extremity joints. In another large-scale study using Southern Pain Prevalence Study data a linear relationship between higher weight and reported severe pain occurring at least monthly was found [3]. While pain was more frequently reported in the lower limbs, moderate to severe pain was also more frequently reported in other parts of the body. They found that as BMI increased, there was a significant increase in the total number of locations of pain reported. These authors suggest a reciprocal relation between pain and obesity.

Rai and Sandell [4] reviewed the relationship between osteoarthritis and obesity, particularly in nonweight-bearing joints. Their review highlights that adipose tissues are a major source of cytokines, chemokines, and metabolically active mediators associated with inflammation. These metabolic factors seem related to both the initiation and progression of osteoarthritis thus, both biomechanical and biochemical factors contribute to pain.

Behavioral and emotional factors may also play a role in obesity and pain. Depressed people are often sedentary, which is associated with obesity and may exacerbate chronic pain. In a recent longitudinal study, a reciprocal link between depression and obesity was found [5] with obesity increasing the risk of depression and depression predicting the development of obesity. Anxiety is often characterized by avoidance behaviors, including restricting life activities. A recent meta-analysis by Gariepy et al. [6] found a positive association between obesity and anxiety in adults. The association was stronger between severe obesity (BMI ≥ 35) and anxiety than for moderate obesity (BMI = 30–35).

Increasingly, bariatric surgery is being considered as a treatment option for severe obesity. Little work to date has focused on the relationship between weight and pain among patients undergoing gastric bypass surgery for weight loss. Three studies have looked at musculoskeletal pain located in the lower extremities and lumbar spine in bariatric surgery populations. They have all found improvement in reported pain following weight loss [7–9]. One study evaluated pain at both weight-bearing and nonweight-bearing musculoskeletal sites both before and then 6–12 months post weight loss surgery [10]. These results were then compared to historical controls. Findings indicated significant improvement of symptoms across all sites with change in BMI the primary factor impacting reported pain. However, these studies have not addressed the larger impact of pain on emotional and physical functioning including functional impairments. It is well established that pain consists of sensory, affective, and cognitive components. These components interact to contribute to emotional and functional impairments.

The present study sought to investigate relations between weight, pain, functional impairment, depression, and anxiety in patients undergoing consideration for bariatric surgery. The primary aim was to better characterize the psychosocial impact of pain on this specific population.

## 2. Methods

One hundred and fifteen patients scheduled to undergo weight loss surgery at a large southeastern academic medical center completed the Brief Pain Inventory (BPI), the Center for Epidemiological Studies 10-item Depression scale (CESD-10), and the Beck Anxiety Inventory (BAI) at the time of their presurgical psychosocial evaluation. Participants were weighed at their clinic visit by the bariatric surgery team prior to surgery. 

Data were collected as part of routine clinical care and IRB approval was attained in order to report the data in aggregate for the purposes of this study.

### 2.1. Measures

The BPI is a 15-item self-report questionnaire, which assesses pain severity and the impact of pain on daily functioning [11]. Each item is ranked from 0–10. The three dimensions of pain severity assessed during the last 24 hours are the worst pain, average pain, and current pain (0 = no pain to 10 = pain as bad as you can imagine). Pain interference items include sleep, work, walking ability, and enjoyment of life (0 = does not interfere to 10 = completely interferes). The BPI has been widely used to assess chronic pain across a variety of conditions, including low back pain [12, 13], arthritis [13], neuropathy [14], and fibromyalgia [15]. Good reliability (coefficient *α* > 0.70) and validity have been demonstrated across multiple pain conditions [13]. 

The CESD-10 is a 10-item self-report questionnaire, ranging from 0–30, which assesses symptoms of depression, including depressed mood, happiness, and lethargy [16]. Any score ≥10 is considered clinically significant. The CESD-10 demonstrates good convergent validity (e.g., negative correlation with positive affect and positive correlation with poor health) and test-retest reliability. It has previously been used in a number of studies that looked at the relationship between obesity and depression [17, 18]. In this study the CESD-10 was found to be a highly reliable measure (*α* = .75).

The BAI is a 21-item self-report questionnaire used for measuring the severity of an individual's anxiety. It assesses common symptoms of anxiety experienced over the past week, including numbness and tingling, shortness of breath, and fear of the worst happening. Items are ranked on a 4-point scale for a maximum total score of 63. Scores are group into minimal (0–7), mild (8–15), moderate (16–25), and severe (26–63) levels of anxiety. Symptoms can be grouped into two groups of somatic and cognitive complaints. The BAI is widely used for assessing clinical anxiety with demonstrated robust psychometric properties [19]. In this study it was found to be a highly reliable measure (*α* = .89).

### 2.2. Data Analyses

Descriptive statistics were calculated for all study variables. Correlational analyses were utilized to examine relations between main study variables of weight and pain, physical activity, mood, walking ability, relationship impairment, enjoyment of life, anxiety, and depression. Theoretically, anxiety and depression are highly related constructs. As such, separate analyses examined the relation between mental health variables, weight, and pain using simultaneous multiple regression models. These models were utilized to examine the predictive relationship between weight, anxiety, depression, and pain on average after accounting for covariates of age and gender. Last, post hoc analyses utilizing Baron and Kenny's [20] four-step approach tested anxiety as a mediator of the relation between weight and pain.

## 3. Results

Eighty percent of the sample was female, 63% was Caucasian (37% African American) with a mean age of 46.6 (SD = 12.7). The mean of the pain-on-average ratings from the BPI was 4.7 (SD = 2.7), the mean of the CESD-10 scores was 7.0 (SD = 4.7) and the mean of the BAI scores was 7.6 (SD = 8.1). The average BMI of the sample was 50.7 (SD = 11.6) and the mean presurgical weight was 310.2 (SD = 76) pounds.  Table 1 presents means, standard deviations, and ranges of the main study variables.

 One item from the pain severity dimension on the BPI was selected (pain on average) along with five pain interference items thought to be most relevant to the multidimensional experience of pain. Correlational analyses were run between weight and the selected items (see Table 2). Higher presurgical weight was associated with higher pain-on-average ratings (*r*(108) = .23, *P* = 0.02), higher functional impairment due to pain across the domains of physical activity (*r*(114) = .19, *P* = 0.04), mood (*r*(113) = .19, *P* = 0.05), walking ability (*r*(114) = .26, *P* = 0.005), relationships (*r*(115) = .23, *P* = 0.02), and enjoyment of life (*r*(115) = .29, *P* = 0.002). Higher presurgical weight was associated with higher BAI scores (*r*(82) = .22, *P* = 0.05), but weight was not related to depression (*r*(87) = −.09, *P* = 0.39).

Simultaneous multiple regressions were then used to evaluate the relation between pain, weight, depression, and anxiety. First the relation between the independent variables weight and the dependent variable pain was examined. Age and gender served as covariates. The independent variable and covariates were entered into the regression at the same time in order to examine the unique predictive variance above all other variables.  Table 3 displays the results for the simultaneous regression for weight predicting pain on average after accounting for covariates age and gender. Increased weight was related to higher levels of pain even after accounting for variance explained by age and gender. 

Second, the relation between the independent variables weight and depression and the dependent variable pain was examined. Age and gender served as covariates.  Table 4 displays the results for the simultaneous regression for weight and depression predicting variance in average pain after accounting for gender and age. Both weight and depression were independently significant predictors of pain after accounting for gender and age such that increased levels of depression and weight were related to increased pain.

Finally, the relation between the independent variables weight and anxiety and the dependent variable pain was examined. Age and gender served as covariates.  Table 5 displays the results for the simultaneous regression for weight and anxiety predicting variance in average pain after accounting for gender and age. Anxiety was a significant predictor of pain after accounting for gender and age such that increased levels of anxiety were related to increased pain. Weight no longer significantly predicted pain on average after accounting for the relation between anxiety and pain; consequently, anxiety serves as a full mediator of the relation between weight and pain (note: all conditions of mediation were met as weight was a significant predictor of pain). 

**Figure 1:**
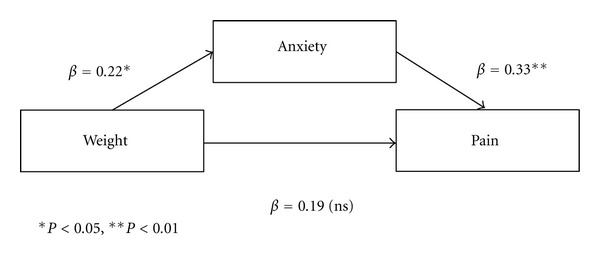
Path diagram with anxiety as mediator of weight and pain.

As Figure 1 illustrates, the standardized regression coefficient between weight and pain was nonsignificant when controlling for anxiety.

## 4. Discussion

This study examined the roles of psychosocial factors in the association between weight and pain. Similarly to previously published findings, higher presurgical weight was associated with higher pain-on-average ratings in this sample of bariatric surgery candidates. In addition, higher presurgical weight was associated with increased functional impairment due to pain across multiple domains including physical activity, mood, walking ability, relationships, and enjoyment of life. Of interest, the strongest associations were found between higher presurgical weight and difficulties in walking ability and enjoyment of life suggesting functional limitations affect both physical and emotional functioning. Bariatric patients often cite functional limitations as a motivating factor in seeking surgery. 

This raises some important clinical implications. Impairment in walking ability may directly impact lifestyle recommendations commonly provided patients including increasing activity levels and regular exercise. This is an area that may require special attention from health care providers who assist patients with behavioral changes as pain while walking may present a specific barrier to increasing activity levels. 

Further, health care providers may want to more specifically target increasing enjoyable life activities in this patient population as there may be benefits for both pain and the psychosocial concomitants of pain. As previously stated, emotional and behavioral factors play a key role in the chronic conditions of both pain and obesity. 

 Little research to date has been done exploring functional impairment due to pain before and after weight loss surgery. Future studies are needed to better determine the most common etiologies of presurgical pain and to determine whether postsurgical weight loss is associated with significant improvement in pain and functional impairment. 

A number of studies have explored the relation between obesity and depression. This study found that both increased weight and depression independently predicted pain. This finding supports the direct effect of both weight and depression on the experience of pain. An unexpected finding from the current study was the association of higher weight with anxiety, but not with depression. This is surprising as depression is a common comorbidity in patients seeking weight loss surgery [21]. However, this population was already restricted to an obese population and thus there may have not been enough weight variation in the sample to see an effect. 

Anxiety in obesity has been much less studied and reported in the literature. A recent study evaluated bariatric patients for depression and anxiety prior to surgery as well as 6–12 months and 24–36 months postoperatively. They found a significant decrease in point prevalence of depressive disorders but not for anxiety disorders after surgery [22]. This suggests that anxiety plays a unique role in lives of an obese population that may persist even after weight loss. 

This study found that anxiety was predictive of pain. In fact, unlike depression and weight, which were found to be independent predictors of pain, weight no longer predicted pain after accounting for the relation between anxiety and pain. Findings indicate that anxiety serves as a full mediator of the relation between weight and pain. This suggests that anxiety has a unique contribution to the obesity and pain relationship in that it exerts a significant indirect effect between weight and pain. As previously noted, anxiety is often accompanied by avoidance behaviors. In addition, pain frequently interferes with life activities. Further studies are needed to explore this unique relation and the effects of anxiety on functioning in obese patients. Findings also suggest that clinicians may want to more carefully evaluate anxiety in presurgical patients, particularly as it relates to pain and functioning.

Anxiety in an obese population presents some unique implications. Obesity is associated with a variety of somatic complaints including shortness of breath, heart palpitations, and sweating which are similarly present in anxiety. In addition, obese individuals often complain of social discomfort and associated anxiety related to perceived negative judgment from others. The BAI was designed to measure both somatic and cognitive symptoms of anxiety. Further studies are needed to more clearly characterize the nature of anxiety in an obese population and more specifically in bariatric surgery candidates. 

One limitation of this study is that the etiology of pain was unknown as was whether the pain was present before obesity or vice versa. Previous studies have indicated a bidirectional influence between obesity and pain [3]. Further, no postsurgical data is yet available to evaluate the impact of weight loss on pain and functioning. Pain and obesity commonly cooccur and as yet remain a relatively little studied area. More studies are needed in order to understand and treat these common and significant health concerns to include the impact on functioning and psychosocial factors.

## Figures and Tables

**Table 1:** Means, standard deviations, and ranges of main study variables.

	M (SD)	Range
Age	46.6 (12.7)	16–71
Weight (lbs)	310.2 (76)	187–608
Pain on average	4.7 (2.7)	0–10
Interference general activity	4.4 (3.5)	0–10
Interference mood	3.7 (3.4)	0–10
Interference walking ability	5.0 (3.5)	0–10
Interference relationships	2.7 (3.0)	0–10
Interference enjoyment of life	4.5 (3.6)	0–10
Depression	7.0 (4.7)	0–21
Anxiety	7.6 (8.1)	0–45

**Table 2:** Relation between presurgical weight and pain and psychosocial factors.

	*β* value	*P* value
Pain on average	.23	0.02
Physical activity	.19	0.04
Mood	.19	0.05
Walking ability	.26	0.005
Relationship impairment	.23	0.02
Enjoyment of life	.29	0.002
BAI	.22	0.05
CESD-10	−.09	0.39

**Table 3:** Weight predicting pain. Model: simultaneous regression predicting brief pain inventory average pain.

	*β*	*b*	SE	*P* value
Age	.26	.05	.02	0.008
Gender	.10	.69	.69	0.32
Weight	.33	.01	.00	0.002

Model *R* ^2^ = .12.

**Table 4:** Weight and depression predicting pain. Model: simultaneous regression predicting brief pain inventory average pain.

	*β*	*b*	SE	*P* value
Age	.23	.05	.02	0.05
Gender	.09	.78	.96	0.42
Weight	.32	.01	.00	0.007
CESD-10	.41	.24	.07	0.000

Model *R* ^2^ = .25.

**Table 5:** Weight and anxiety predicting pain. Model: simultaneous regression predicting brief pain inventory average pain.

	*β*	*b*	SE	*P* value
Age	.19	.04	.03	0.14
Gender	.01	.08	.99	0.94
Weight	.19	.00	.00	0.16
BAI	.33	.11	.04	0.01

Model *R* ^2^ = .20.

## References

[1] Stone AA, Broderick JE (2012). Obesity and pain are associated in the United States. *Obesity*.

[2] Andersen RE, Crespo CJ, Bartlett SJ, Bathon JM, Fontaine KR (2003). Relationship between body weight gain and significant knee, hip, and back pain in older Americans. *Obesity Research*.

[3] Hitt HC, McMillen RC, Thornton-Neaves T, Koch K, Cosby AG (2007). Comorbidity of obesity and pain in a general population: results from the Southern pain Prevalence Study. *Journal of Pain*.

[4] Rai MF, Sandell LJ (2011). Inflammatory mediators: tracing links between obesity and osteoarthritis. *Critical Reviews in Eukaryotic Gene Expression*.

[5] Luppino FS, De Wit LM, Bouvy PF (2010). Overweight, obesity, and depression: a systematic review and meta-analysis of longitudinal studies. *Archives of General Psychiatry*.

[6] Gariepy G, Nitka D, Schmitz N (2010). The association between obesity and anxiety disorders in the population: a systematic review and meta-analysis. *International Journal of Obesity*.

[7] Peltonen M, Lindroos AK, Torgerson JS (2003). Musculoskeletal pain in the obese: a comparison with a general population and long-term changes after conventional and surgical obesity treatment. *Pain*.

[8] McGoey BV, Deitel M, Saplys RJF, Kliman ME (1990). Effect of weight loss on musculoskeletal pain in the morbidly obese. *Journal of Bone and Joint Surgery Series B*.

[9] Melissas J, Volakakis E, Hadjipavlou A (2003). Low-back pain in morbidly obese patients and the effect of weight loss following surgery. *Obesity Surgery*.

[10] Hooper MM, Stellato TA, Hallowell PT, Seitz BA, Moskowitz RW (2007). Musculoskeletal findings in obese subjects before and after weight loss following bariatric surgery. *International Journal of Obesity*.

[11] Cleeland CS, Osoboa D (1991). Pain assessment in cancer. *Effect of Cancer on Quality of Life*.

[12] Gammaitoni AR, Galer BS, Lacouture P, Domingos J, Schlagheck T (2003). Effectiveness and safety of new oxycodone/acetaminophen formulations with reduced acetaminophen for the treatment of low back pain. *Pain Medicine*.

[13] Keller S, Bann CM, Dodd SL, Schein J, Mendoza TR, Cleeland CS (2004). Validity of the brief pain inventory for use in documenting the outcomes of patients with noncancer pain. *Clinical Journal of Pain*.

[14] Gimbel JS, Richards P, Portenoy RK (2003). Controlled-release oxycodone for pain in diabetic neuropathy: a randomized controlled trial. *Neurology*.

[15] Arnold LM, Rosen A, Pritchett YL (2005). A randomized, double-blind, placebo-controlled trial of duloxetine in the treatment of women with fibromyalgia with or without major depressive disorder. *Pain*.

[16] Andresen EM, Malmgren JA, Carter WB, Patrick DL (1994). Screening for depression in well older adults: evaluation of a short form of the CES-D. *American Journal of Preventive Medicine*.

[17] Vogelzangs N, Kritchevsky SB, Beekman ATF (2010). Obesity and onset of significant depressive symptoms: results from a prospective community-based cohort study of older men and women. *Journal of Clinical Psychiatry*.

[18] Johnston E, Johnson S, McLeod P, Johnston M (2004). The relation of body mass index to depressive symptoms. *Canadian Journal of Public Health*.

[19] Beck AT, Epstein N, Brown G, Steer RA (1988). An inventory for measuring clinical anxiety: psychometric properties. *Journal of Consulting and Clinical Psychology*.

[20] Baron RM, Kenny DA (1986). The moderator-mediator variable distinction in social psychological research: conceptual, strategic, and statistical considerations. *Journal of Personality and Social Psychology*.

[21] Dixon JB, Dixon ME, O’Brien PE (2003). Depression in association with severe obesity: changes with weight loss. *Archives of Internal Medicine*.

[22] de Zwaan M, Enderle J, Wagner S (2011). Anxiety and depression in bariatric surgery patients: a prospective, follow-up study using structured clinical interviews. *Journal of Affective Disorders*.

